# Faba Bean (*Vicia faba* L.) Nodulating Rhizobia in Panxi, China, Are Diverse at Species, Plant Growth Promoting Ability, and Symbiosis Related Gene Levels

**DOI:** 10.3389/fmicb.2018.01338

**Published:** 2018-06-20

**Authors:** Yuan X. Chen, Lan Zou, Petri Penttinen, Qiang Chen, Qi Q. Li, Chang Q. Wang, Kai W. Xu

**Affiliations:** ^1^College of Resources, Sichuan Agricultural University, Chengdu, China; ^2^Zhejiang Provincial Key Laboratory of Carbon Cycling in Forest Ecosystems and Carbon Sequestration, School of Environmental & Resource Sciences, Zhejiang Agriculture & Forestry University, Lin'an, China; ^3^Ecosystems and Environment Research Programme, Faculty of Biological and Environmental Sciences, University of Helsinki, Helsinki, Finland

**Keywords:** faba bean, rhizobia, genetic diversity, multilocus sequence analysis, symbiosis gene, lateral gene transfer

## Abstract

We isolated 65 rhizobial strains from faba bean (*Vicia faba* L.) from Panxi, China, studied their plant growth promoting ability with nitrogen free hydroponics, genetic diversity with clustered analysis of combined ARDRA and IGS-RFLP, and phylogeny by sequence analyses of 16S rRNA gene, three housekeeping genes and symbiosis related genes. Eleven strains improved the plant shoot dry mass significantly comparing to that of not inoculated plants. According to the clustered analysis of combined ARDRA and IGS-RFLP the isolates were genetically diverse. Forty-one of 65 isolates represented *Rhizobium anhuiense*, and the others belonged to *R. fabae, Rhizobium vallis, Rhizobium sophorae, Agrobacterium radiobacter*, and four species related to *Rhizobium* and *Agrobacterium*. The isolates carried four and five genotypes of *nifH* and *nodC*, respectively, in six different *nifH*-*nodC* combinations. When looking at the species-*nifH*-*nodC* combinations it is noteworthy that all but two of the six *R. anhuiense* isolates were different. Our results suggested that faba bean rhizobia in Panxi are diverse at species, plant growth promoting ability and symbiosis related gene levels.

## Introduction

Legumes like faba bean (*Vicia faba* L.) and rhizobial bacteria can form a symbiotic relationship in which the legume host provides the rhizobia with nutrients and niche while rhizobia provide the host with fixed atmospheric dinitrogen in the form of ammonia. Owing to symbiosis, legumes can act as pioneer plants in nitrogen deficient areas and improve soil fertility (Graham and Vance, 2003; Gentzbittel et al., 2015). Nitrogen fertilization affects the environment; however, applying biological N fixation (BNF) has some advantages over synthetic N fertilizers. If incorporated into the soil, legumes do not acidify the soil like ammonium-based fertilizers (Crews and Peoples, 2004). Unlike the production of synthetic N fertilizers, BNF does not rely on non-renewable energy sources (Crews and Peoples, 2004).

In legumes, nitrogen fixation takes place in a specific root or stem organ called nodule. The formation of plant growth promoting symbiosis requires that the legume and the rhizobia are compatible, and that the rhizobia fix nitrogen efficiently. Inoculating the legume with suitable rhizobia increases growth when compatible rhizobia are not present or when the compatible rhizobia are not efficient (Thilakarathna and Raizada, 2017).

Faba bean, a grain legume grown worldwide, is a good resource of protein, starch, cellulose and minerals. Its high yield and great adaption to different environments makes faba bean very popular among farmers, feed and food manufacturers (Haciseferoǧullar et al., 2003). Moreover, the capacity for biological nitrogen fixation with rhizobial bacteria makes faba bean a renewable resource for sustainable agriculture (Köpke and Nemecek, 2010). Thus, it is common that faba bean is grown as an intercrop or in rotation with non-legume plants (Song et al., 2007; Mei et al., 2012). However, in China faba bean frequently receive synthetic N fertilizer, resulting in over fertilization (Li et al., 2016).

Panxi region in Sichuan, southwestern China, is on the western margin of Yangtze Block, between Tibet Plateau, Yunnan-Guizhou Plateau and Sichuan basin. Panxi is within the South-West China mountains biodiversity hotspot (Wu et al., 2006; www.cepf.net/resources/hotspots/Asia-Pacific/Pages/Mountains-of-Southwest-China.aspx). Mountains occupy 80% of the total area of Panxi and the altitude differences in this area reach 5,600 m. Panxi receives plenty of rainfall and strong solar radiation, and the climate ranges from southern Asian semitropical climate to northern temperate climate with xerothermic climate as the main characteristic of the arid-hot river valley area. Faba bean is one of the main crops in Panxi. Cultivation relies on seeds produced by farmers themselves. N fertilizers would be unnecessary if the soils hosted compatible, plant growth promoting rhizobia.

The species range of rhizobia nodulating legumes in Panxi differs from that in other parts of China. For example, in Panxi *Leucaena leucocaphala* and *Pueraria lobate* were mostly nodulated by *Ensifer* and *Rhizobium* strains, respectively, while in subtropical China *L. leucocaphala* was nodulated by *Mesorhizobium* strains and in other parts of Sichuan *P. lobate* by *Bradyrhizobium* strains (Chen et al., 2004; Wang et al., 2006; Xie et al., 2009; Xu et al., 2013). Since faba bean rhizobia in Panxi have not been studied systematically prior to this study, our aim was to assess if faba bean rhizobia in the area were diverse and unique. Thus, we isolated rhizobia from faba bean growing in the special arid-hot environment of Panxi in diverse soil types, and studied their plant growth promoting ability, genetic diversity and phylogeny based on molecular methods.

## Materials and methods

### Isolation of strains

**Figure 1:**
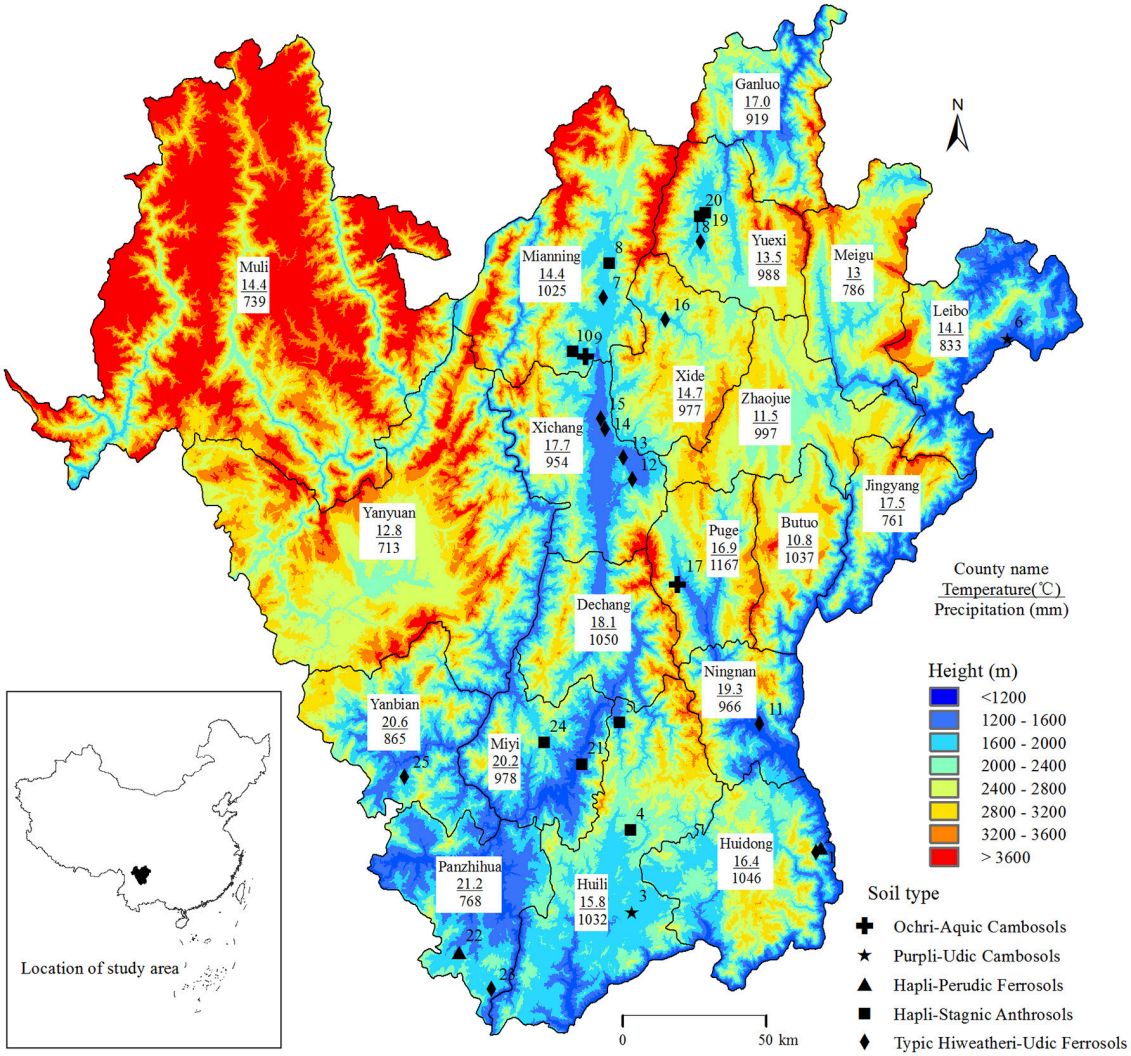
The 25 sampling sites in Panxi. The location of Panxi in China is shown in the inset. The maps were drawn using ArcGIS 10.0 software. Temperature and precipitation refer to the average annual values.

Local variety faba bean samples were taken in 25 sites in Panxi, Sichuan, China (Figure 1) to collect root nodules. Nodules were surface sterilized in 95% ethanol for 3 min and 0.1% HgCl_2_ for 5 min, followed by rinsing six times in sterile distilled water. The sterilized nodules were crushed individually and streaked on yeast extract mannitol (YEM) medium (Vincent, 1970) containing 25 mg L^−1^ congo red at 28°C. The purified strains were stored on YEM slants at 4°C for short term and in 25% glycerol at −80°C for long term storage.

### Nodulation assays

The nodulation ability and symbiotic efficiency of the isolates was tested on the local faba bean (*V. faba* L.) cultivar Hanyuan dabaidou. Seeds of faba bean were immersed in 95% ethanol for 5 min, rinsed for 5 min with 0.2% mercury bichloride (HgCl_2_) and 8 times (10 min per time) with sterilized water. After surface sterilization, the seeds were soaked in sterilized water overnight to soften the thick and hard seed coat. The seeds were transferred on 0.5% water-agar for germination. The seedlings were transplanted in sterile 250 ml infusion bottles containing Jensen's solution (Vincent, 1970) in all inoculation assays. The seedlings were inoculated with 1.5 ml of the culture containing ca 10^9^ bacterial cells per milliliter and grown under a 16 h light and 8 h dark regime at 25°C in greenhouse. The assays were done in triplicate with one seedling per bottle, including the uninoculated controls. After 50 days, the plants were harvested and the numbers of nodules and the plant shoot dry mass were measured. One-way analysis of variance with a least significant difference (LSD) analysis (*P* = 0.05) was done using Excel 2010 (Microsoft, Redmond, USA) and SPSS 17.0 (SPSS Inc., Chicago, USA).

### PCR-RFLP and CACAI

Total DNA was extracted by GUTC (Guanidinium-Tris-CDTA buffer with celite) method (Terefework et al., 2001) from purified bacteria. 16S rDNA and intergenic spacer region (IGS) of the strains were amplified for restriction fragment length polymorphism analysis. Primer pairs P1, P6 and pHr(F), p23SR01(R) (Table 1) were used for polymerase chain reaction (PCR) amplification. Amplification products (5 μl) were digested separately by four restriction enzymes *Hin*fI, *Taq*I, *Msp*I, and *Hae*III following the manufacturer's instructions (Fermentas, EU). The fragments were separated by gel electrophoreses in 2% agarose with 0.5 μg ml^−1^ ethidium bromide at 80 V for 3 h and photographed. Amplified ribosomal DNA restriction analysis (ARDRA) and IGS-RFLP were done by combining the results from the four restrictions. Clustered analysis of combined ARDRA and IGS-RFLP (CACAI) was conducted by UPGM clustering algorithm in the NTSYS program (Rohlf, 1990).

**Table 1:** PCR primers and reaction procedures applied in this study.

**Gene**	**Primers**	**Reaction procedure**	**References**
16S rDNA	P1:5′-AGAGTTTGATCCTGGCTCAGAACGAACGCT-3′; P6: 5′- TACGGCTACCTTGTTACGACTTCACCCC-3′	92°C for 3 min, 30 cycles of 94°C for 1 min, 58°C for 1 min, 72°C for 2 min, final extension for 72°C 8 min	Tan et al., 1997
IGS	pHr (F):5′-TGCGGCTGGATCACCTCCTT-3′; p23SR01(R):5′-GGCTGC TTCTAAGCCAAC-3′	92°C for 3 min, 30 cycles of 94°C for 1 min, 60°C for 1 min, 72°C for 2 min, final extension for 72°C 8 min	Navarro et al., 1992; Massol-Deya et al., 1995
*atpD*	atpD255F: 5′-GCTSGGCCGCATCMTSAACGTC-3′; atpD782R: 5′-GCCGACACTTCMGAACCNGCCTG-3′	95°C for 2 min; 30 cycles of 94°C for 45 s, 59°C for 1 min, 72°C for 1.5 min; final extension 72°C for 10 min	Vinuesa et al., 2005
*glnII*	glnII12F:YAAGCTCGAGTACATYTGGCT; glnII689R: TGCATGCCSGAGCCGTTCCA	95°C for 5 min; 30 cycles of 94°C for 1 min, 58°C for 1 min, 72°C for 1 min; final extension 72°C for 10 min	Vinuesa et al., 2005
*recA*	recA 41F: 5′-TTCGGCAAGGGMTCGRTSATG-3′; recA 640R: 5′-ACATSACRCCGATCTTCATGC-3′	95°C for 5 min; 30 cycles of 94°C for 1 min, 58°C for 1 min, 72°C for 1 min; final extension 72°C for 10 min	Vinuesa et al., 2005
*nifH*	nifHctg:5′-CTCATCGTCGGCTGTGACCC-3′; nifHI: 5′-AGCATGTCYTCSAGYTCNTCCA-3′	95°C for 3 min; 40 cycles of 94°C for 1 min, 59°C for 1 min, 72°C for 1 min; final extension 72°C for 5 min	Laguerre et al., 2001; Gurkanli et al., 2014
	nifH1F:5′-GTCTCCTATGACGTGCTCGG-3′; nifH1R:5′-GCTTCCATGGTGATCGGGGT-3′	94°C for 3 min; 30 cycles of 94°C for 30 s, 58°C for 30 s, 72°C for 1 min; final extension 72°C for 5 min	Rivas et al., 2002
*nodC*	nodC540: 5′-TGATYGAYATGGARTAYTGGYT-3′; nodC1160: 5′-CGYGACAGCCANTCKCTATTG-3′	95°C for 5 min; 30 cycles of 94°C for 1 min, 55°C for 1 min, 72°C for 1 min; final extension 72°C for 10 min	Sarita et al., 2005
	nofCf: 5′-GCTGCCTATGCAGACGATG-3′; nodCr: 5′-GGTTACTGGCTTTCATTTGGC-3′	94°C for 5 min; 30 cycles of 94°C for 1 min, 55°C for 1 min, 72°C for 3 min; final extension 72°C for 7 min	Moschetti et al., 2005

### Sequencing of housekeeping and symbiotic genes

According to the results of CACAI, representative strains were selected for sequencing of housekeeping and symbiotic genes. To facilitate the comparison of faba bean nodulating diversity in Panxi and other parts of Sichuan, we applied the same methods as in our earlier study on rhizobia from Sichuan hilly areas (Xu et al., 2015). 16S rDNA was amplified as described above. Three housekeeping genes *atpD, glnII*, and *recA* and two symbiotic genes *nifH* and *nodC* were amplified as described in Table 1. The PCR products were sequenced directly at BGI Tech (Shenzhen, China). Sequences have been deposited to NCBI (National Center for Biotechnology Information research database) nucleotide database under the accession numbers of KU947312-KU947400.

The sequences of the housekeeping and symbiotic genes were compared with sequences in NCBI, and the 16S rDNA sequences were compared with sequences in EzTaxon (http://www.ezbiocloud.net/) using BLASTN. Phylogenetic analyses of sequences from our isolates and reference sequences from databases were done using a Neighbor-Joining method in MEGA 6.0 (Tamura et al., 2013) with 1,000 bootstrapped replicates. Genospecies were defined by multilocus sequence analysis (MLSA) using concatenated sequence of three housekeeping genes applying 97% average nucleotide identity as the threshold (Cao et al., 2014).

## Results

### Nodulation, plant growth promoting ability, and genetic diversity of faba bean isolates

We isolated 65 strains from root nodules of faba bean growing in Panxi, China (Table 2). All but two of the strains formed nodules on the roots of faba bean with the average nodule numbers ranging from 3.0 to 98.5 per plant. No nodules were detected on the roots of the uninoculated plants (Table 2). The plant growth promoting ability of the isolates was assessed by measuring the dry masses of the inoculated plants. The eleven strains that significantly increased the plant shoot dry mass (*p* < 0.05) were considered as potential inoculant strains (Table 3).

**Table 2:** Rhizobial isolates from faba bean in Panxi, their genetic and symbiotic characteristics and phylogenetic affiliation.

**Isolate^a^**	**Sampling site^b^**	**16S rDNA RFLP genotype^c^**	**IGS RFLP genotype^c^**	**CACAI genotype**	**CACAI group^d^**	**MLSA^e^**	**Plant shoot dry mass (g plant^−1^)^f^**	**No. of nodules per plant**
SCAUf129	21	a	1	1	A		1.593 ± 0.340↑^*^	34.3
SCAUf141	17	a	17	5	A		1.523 ± 0.020↑^*^	41.7
SCAUf142	4	a	1	1	A		1.457 ± 0.247↑^*^	93.0
SCAUf110	11	a	21	9	B		1.397 ± 0.301↑^*^	18.7
SCAUf126	12	a	1	1	A		1.377 ± 0.074↑^*^	25.0
SCAUf118	7	a	1	1	A		1.375 ± 0.025↑^*^	62.0
SCAUf114	12	a	1	1	A		1.327 ± 0.186↑^*^	40.3
SCAUf122	14	a	1	1	A		1.287 ± 0.156↑^*^	39.7
SCAUf123	9	g	22	24	C		1.283 ± 0.063↑^*^	43.3
SCAUf113	22	a	1	1	A		1.280 ± 0.265↑^*^	94.3
SCAUf148	16	h	20	25	D		1.267 ± 0.201↑^*^	52.0
**SCAUf131**	25	a	1	1	A	*R. anhuiense* CCBAU 23252^T^ (98.5%)	1.215 ± 0.365	98.5
SCAUf125	18	a	23	10	E		1.135 ± 0.095	61.5
SCAUf136	9	a	1	1	A		1.130 ± 0.160	58.7
SCAUf111	21	a	1	1	A		1.127 ± 0.109	72.3
**SCAUf133**	11	a	19	7	B	*R. sophorae* CCBAU 03386^T^ (96.6 %)	1.120 ± 0.210	17.0
SCAUf102	2	a	1	1	A		1.097 ± 0.138	28.3
**SCAUf106**	1	a	5	13	F	*R. vallis* CCBAU 65647^T^ (93.0%)	1.095 ± 0.088	48.0
**SCAUf109**	1	a	4	12	G	*R. vallis* CCBAU 65647^T^ (93.0%)	1.094 ± 0.087	46.7
SCAUf139	4	a	1	1	A		1.087 ± 0.121	40.3
**SCAUf150**	16	h	20	25	D	*A. radiobacter* NCPPB 2437^T^ (97.3%)	1.080 ± 0.131	4.0
SCAUf121	23	a	18	6	A		1.050 ± 0.044	44.7
SCAUf134	15	a	19	7	B		1.017 ± 0.153	27.0
SCAUf115	7	a	1	1	A		0.950 ± 0.410	22.5
**SCAUf87**	19	c	8	19	H	*A. radiobacter* NCPPB 2437^T^ (97.3%)	0.934 ± 0.146	23.0
SCAUf137	23	a	18	6	A		0.933 ± 0.206	35.0
SCAUf101	2	a	1	1	A		0.930 ± 0.021	27.7
SCAUf143	15	a	19	7	B		0.923 ± 0.228	22.5
SCAUf147	15	a	19	7	B		0.893 ± 0.173	22.0
SCAUf146	12	a	1	1	A		0.870 ± 0.150	4.5
**SCAUf99**	20	a	6	14	E	*R. sophorae* CCBAU 03386^T^ (96.6 %)	0.864 ± 0.041	59.7
SCAUf92	1	a	2	8	A		0.856 ± 0.099	23.7
SCAUf132	21	a	1	1	A		0.833 ± 0.231	16.7
SCAUf103	19	f	15	22	I		0.820 ± 0.066	68.0
SCAUf107	13	a	12	4	J		0.815 ± 0.066	3.0
**SCAUf149**	11	i	25	26	K	*A. radiobacter* NCPPB 2437^T^ (97.3%)	0.805 ± 0.155	35.0
CK		–	–	–			0.801 ± 0.139	0.0
SCAUf95	5	a	1	1	A		0.790 ± 0.169	20.0
SCAUf120	17	a	17	5	A		0.790 ± 0.030	25.0
**SCAUf90**	10	a	11	3	B	*R. sophorae* CCBAU 03386^T^ (96.6 %)	0.785 ± 0.051	60.3
**SCAUf94**	8	a	9	16	L	*R. vallis* CCBAU 65647^T^ (93.0%)	0.780 ± 0.060	18.7
**SCAUf86**	13	b	12	17	J	*R. sophorae* CCBAU 03386^T^ (99.3 %)	0.770 ± 0.121	12.7
SCAUf124	14	a	1	1	A		0.753 ± 0.067	36.0
SCAUf138	14	a	1	1	A		0.750 ± 0.057	38.0
SCAUf145	18	a	17	5	A		0.750 ± 0.110	35.5
**SCAUf140**	17	a	18	6	A	*R. anhuiense* CCBAU 23252^T^ (98.9%)	0.743 ± 0.170	23.0
SCAUf117	22	a	1	1	A		0.737 ± 0.177	71.7
**SCAUf127**	22	a	1	1	A	*R. anhuiense* CCBAU 23252^T^ (98.9%)	0.733 ± 0.065	41.3
SCAUf128	9	a	1	1	A		0.707 ± 0.188	13.3
**SCAUf100**	10	e	13	21	M	*R. vallis* CCBAU 65647^T^ (99.0%)	0.689 ± 0.034	11.0
**SCAUf104**	24	f	16	23	I	*R. anhuiense* CCBAU 23252^T^ (99.2%)	0.680 ± 0.082	15.0
SCAUf89	20	a	10	2	B		0.680 ± 0.060	21.3
SCAUf119	4	a	1	1	A		0.667 ± 0.188	20.0
SCAUf135	4	a	1	1	A		0.663 ± 0.114	16.7
**SCAUf105**	3	a	3	11	A	*R. anhuiense* CCBAU 23252^T^ (99.3%)	0.660 ± 0.020	5.0
SCAUf108	3	a	7	15	A		0.650 ± 0.031	6.3
SCAUf116	25	a	1	1	A		0.647 ± 0.189	47.0
SCAUf112	21	a	1	1	A		0.647 ± 0.099	11.7
SCAUf96	5	a	1	1	A		0.643 ± 0.070	21.3
SCAUf88	19	a	2	8	A		0.632 ± 0.073	15.0
**SCAUf144**	4	b	24	18	C	*R. fabae* CCBAU 33202^T^ (99.9 %)	0.605 ± 0.090	24.0
SCAUf97	19	a	1	1	A		0.573 ± 0.032	31.0
**SCAUf91**	6	a	2	8	A	*R. anhuiense* CCBAU 23252^T^ (99.8%)	0.533 ± 0.087	13.0
SCAUf130	23	a	18	6	A		0.532± 0.086	23.0
**SCAUf93**	19	d	14	20	N	*A. radiobacter* NCPPB 2437^T^ (97.3%)	0.532± 0.069	0
SCAUf98	19	a	1	1	A		0.517± 0.059	0

a CK: uninoculated treatment in the symbiotic efficiency test. Representative isolates for sequencing in bold.

b Sampling sites are the same as Figure 1.

c Genotype: the combination of the restriction patterns obtained by enzymes MspI, HaeIII, TaqI, and HinfI.

d CACAI: clustered analysis of combined ARDRA and IGS-RFLP, Groups were defined at 94.5% similarity level.

e MLSA, multilocus sequence analysis of combined recA, atpD, and glnII. The percentages are sequence similarities to the closely related species or the closest type strain.

t Type strain.

f ↑* *Significantly higher shoot dry mass than that in CK treatment according to the LSD test (P = 0.05). Data presented as mean value ± standard deviation (n = 3, except n = 2 for SCAUf93 and SCAUf98)*.

**Table 3:** The *nodC* and *nifH* types in the representative strains isolated from faba bean.

**Representative strain**	***nodC*^a^**	***nifH*^a^**
*R. anhuiense* SCAUf104	*R. fabae*	HRsp1
*R. anhuiense* SCAUf127	*R. fabae*	HRsp1
*R. fabae* SCAUf144	*R. fabae*	HRsp1
*R. sophorae* SCAUf86	*R. fabae*	HRsp2
*R. anhuiense* SCAUf131	*R. fabae*	HRsp2
*R. anhuiense* SCAUf140	*R. laguerreae*	HRsp1
*R. anhuiense* SCAUf105	CRla1	HRsp2
*R. vallis* SCAUf100	CRsp1	HRsp3
*R. anhuiense* SCAUf91	CRsp2	HRsp4
*Rhizobium* sp.I f99	CRsp2	HRsp4
*Rhizobium* sp.I SCAUf90	CRsp2	HRsp4
*Rhizobium* sp.I SCAUf133	CRsp2	HRsp4
*Rhizobium* sp.II SCAUf94	CRsp2	HRsp4
*Rhizobium* sp.II SCAUf106	CRsp2	HRsp4
*Rhizobium* sp.II SCAUf109	CRsp2	HRsp4

a *Closest type strain or clade*.

Amplification of the 16S rDNA gene resulted in an approximately 1,500 bp band from all the isolates. In the 16S rDNA PCR-RFLP, nine fragment pattern types (a-i) were observed: type a included 54 strains, types b, f, and h included two strains each, and types c, d, e, g, and i included one strain each (Table 2).

For the majority of strains, IGS PCR resulted in a single band ranging from 1,900 to 2,200 bp, whereas for strains SCAUf90 and SCAUf99 IGS PCR resulted in two and three bands, respectively (Table 2). The strains were divided to 25 IGS-RFLP types. In the combined analysis of 16S rDNA RFLP and IGS-RFLP (CACAI) the strains were divided into 14 CACAI groups at 94.5% similarity level and 26 CACAI genotypes (Table 2). CACAI group A was the largest group including 40 isolates with CACAI genotypes 1, 5, 6, 8, and 15. Seven of the plant growth promoting strains represented genotype 1, and the other four were assigned to genotypes 5, 9, 24, and 25.

### 16S rDNA phylogeny

**Figure 2:**
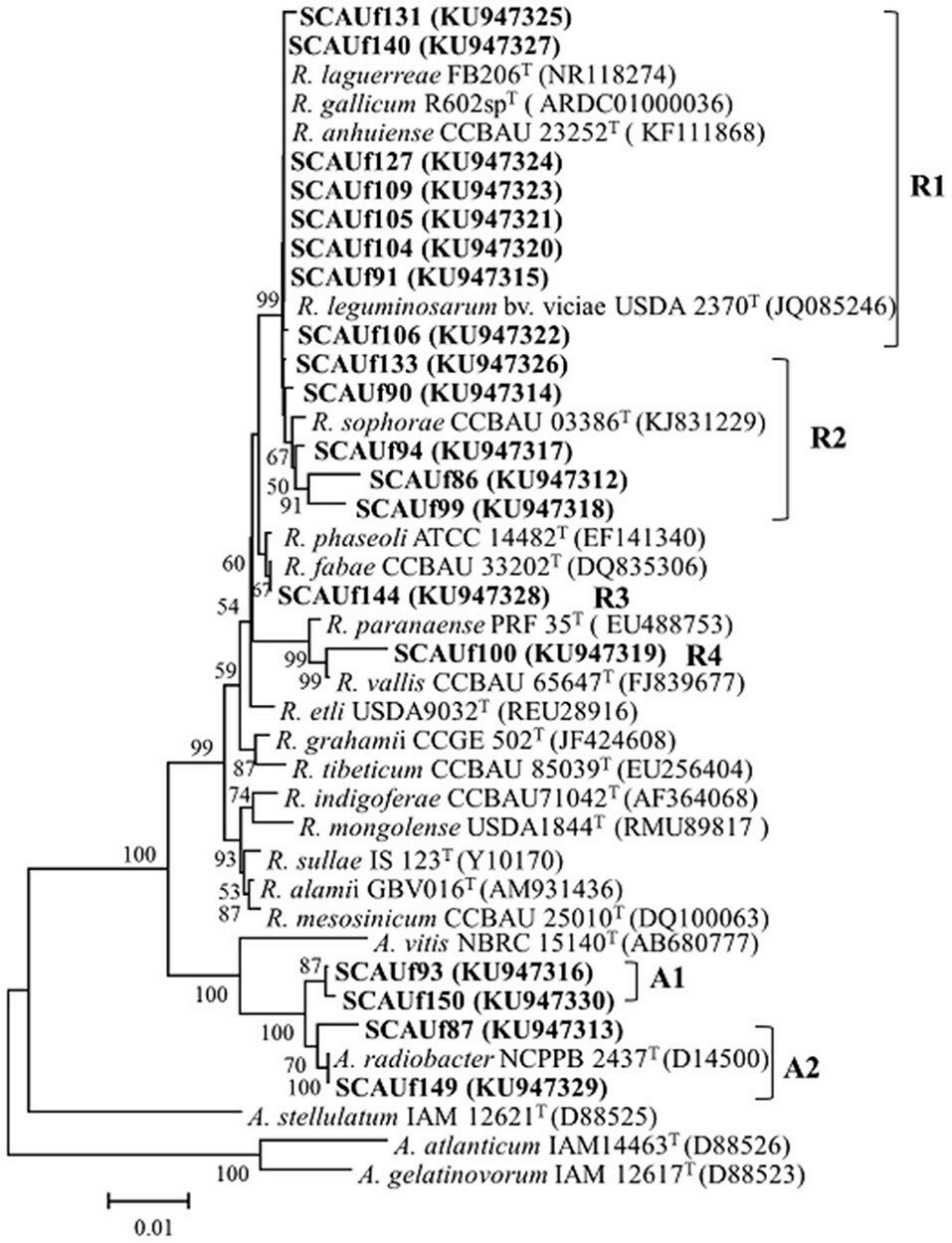
Neighbor-joining tree based on 16S rDNA (1,336 nt) presenting the phylogenetic relationship among the representative strains isolated from faba bean (in bold) and reference strains. Bootstrap values ≥50% are shown on the branches. Genbank accession numbers are in parentheses. Scale bar = 1% substitutions per site. *R, Rhizobium*; *A, Agrobacterium*.

Based on CACAI groups as well as considering the sites of isolation of the strains, 19 representative strains were selected for sequencing. In the 16S rDNA phylogenetic tree (Figure 2), the strains clustered into six distinct clades with the reference strains. Four clades were related to *Rhizobium* (R group) and two to *Agrobacterium* (A group). Four strains clustered with *Agrobacterium radiobacter* type strain with 98.3–99.8% similarities. SCAUf144 clustered with *R. fabae* with 100% similarity, SCAUf100 clustered with *R. vallis* with 98.6% similarity, and SCAUf86, SCAUf90, SCAUf94, SCAUf99, and SCAUf133 clustered with *Rhizobium sophorae* into clade R2 with 97.9–99.9% similarities. The other eight strains clustered into a distinct clade with *R. gallicum, Rhizobium anhuiense, R. laguerreae*, and *Rhizobium leguminosarum* with similarities ranging from 99.8 to 100%.

### Multilocus sequence analysis

**Figure 3:**
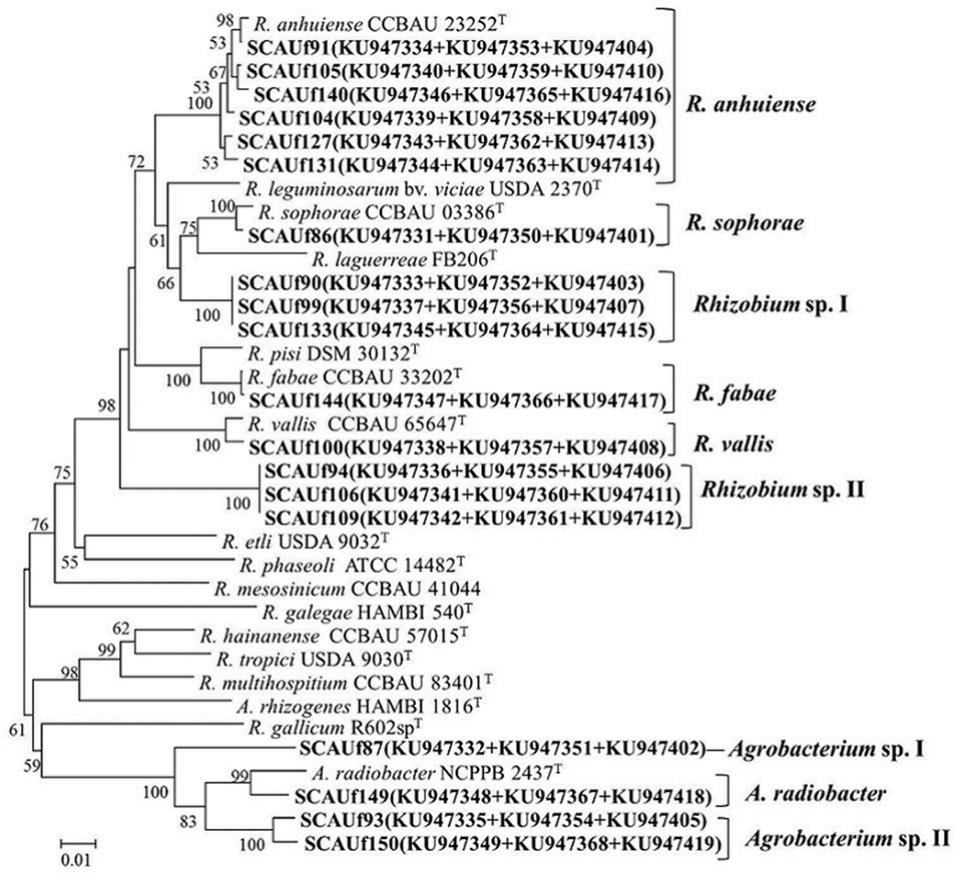
Neighbor-joining tree based on multilocus sequence analysis using concatenated sequence of *atpD* (395 nt), *glnII* (483 nt), and *recA* (344 nt) genes presenting the phylogenetic relationship among the representative strains isolated from faba bean (in bold) and reference strains. Bootstrap values ≥50% are shown on the branches. Genbank accession numbers are in parentheses. Scale bar = 1% substitutions per site. *R, Rhizobium, A, Agrobacterium*.

In the multilocus sequence analysis (MLSA) based on housekeeping genes *atpD, glnII* and *recA*, the 19 representative strains clustered into nine distinct clades related to *Rhizobium* and *Agrobacterium* species (Figure 3). SCAUf86 was 99.3% similar to *R. sophorae* CCBAU 03386^T^, thus assigned as *R*. *sophorae*. SCAUf90, SCAUf99 and SCAUf133 clustered separately and were assigned as *Rhizobium* sp. I, as did SCAUf94, SCAUf106 and SCAUf109 that were assigned as *Rhizobium* sp. II. SCAUf91, SCAUf104, SCAUf105, SCAUf127, SCAUf131, and SCAUf140 were 98.5–99.8% similar to *R. anhuiense* type strain, thus assigned as *R*. *anhuiense* strains. SCAUf144 and SCAUf100 clustered with *R. fabae* CCBAU 33202^T^ and *R. vallis* CCBAU 65647^T^, respectively, thus assigned as *R*. *fabae* and *R*. *vallis*, respectively. As in 16S rDNA analysis, four strains clustered with *Agrobacterium* in the MLSA. Because no *glnII* sequences of the relevant *Agrobacterium* type strains except *A. radiobacter* type strain were available in the GenBank sequence database, the relationships between *Agrobacterium* strains were studied based on non-type strains (Supplementary Figure S1). SCAUf87 clustered separately, and was assigned as *Agrobacterium* sp. I. SCAUf93 and SCAUf150 clustered separately and were assigned as *Agrobacterium* sp. II. SCAUf149 was 97.3% similar to *A. radiobacter* NCPPB 2437^T^ with, thus assigned as *A. radiobacter*.

### Diversity of symbiosis genes

**Figure 4:**
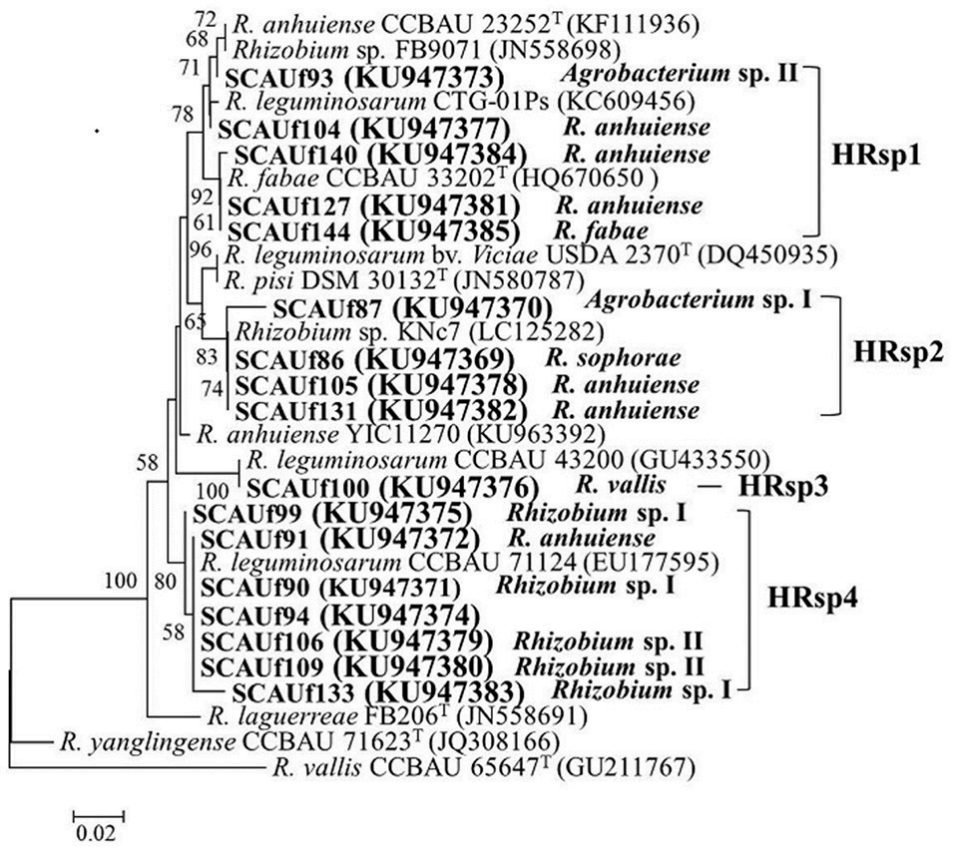
Neighbor-joining tree based on *nifH* (329 nt) gene of 17 representative strains isolated from faba bean (in bold) and reference strains. Genbank accession numbers are in parentheses. Bootstrap values ≥50% are shown on the branches. Scale bar = 2% substitutions per site. *R, Rhizobium*.

For both *nifH* and *nodC* amplification was not successful with one primer pair only, possibly due to differences in primer binding sites. Approximately 700 bp fragments were obtained using primer pair nifHctg/nifHI (13 representative strains), and 400 bp products using primer pair nifH1F/ nifH1R (SCAUf87, SCAUf105, SCAUf133, SCAUf140). Amplification of *nifH* from SCAUf149 and SCAUf150 was not successful. Seventeen strains clustered into four clades in the *nifH* phylogenetic tree (Figure 4, Table 3). The *nifH* of *Agrobacterium* sp. II SCAUf93 and *R. anhuiense* SCAUf104 were 99.7 and 99.6%, respectively, similar to that of *R. anhuiense* CCBAU 23252^T^. The *nifH* of *R. anhuiense* SCAUf127 and *R. fabae* SCAUf144 were 100% similar to that of *R*. *fabae* type strain. The *nifH* of *R. sophorae* SCAUf86, *R. anhuiense* SCAUf105 and SCAUf131 clustered with that of *R. leguminosarum* USDA 2370^T^ with 98.4% similarity. *R. vallis* SCAUf100 clustered with *R. leguminosarum* CCBAU 43200 with 100% similarity. The strains *R. anhuiense* SCAUf91, *Rhizobium* sp. I SCAUf90, *Rhizobium* sp. II SCAUf94, SCAUf106 and SCAUf109 carried *nifH* 100% similar to that of *R. leguminosarum* CCBAU 71124.

**Figure 5:**
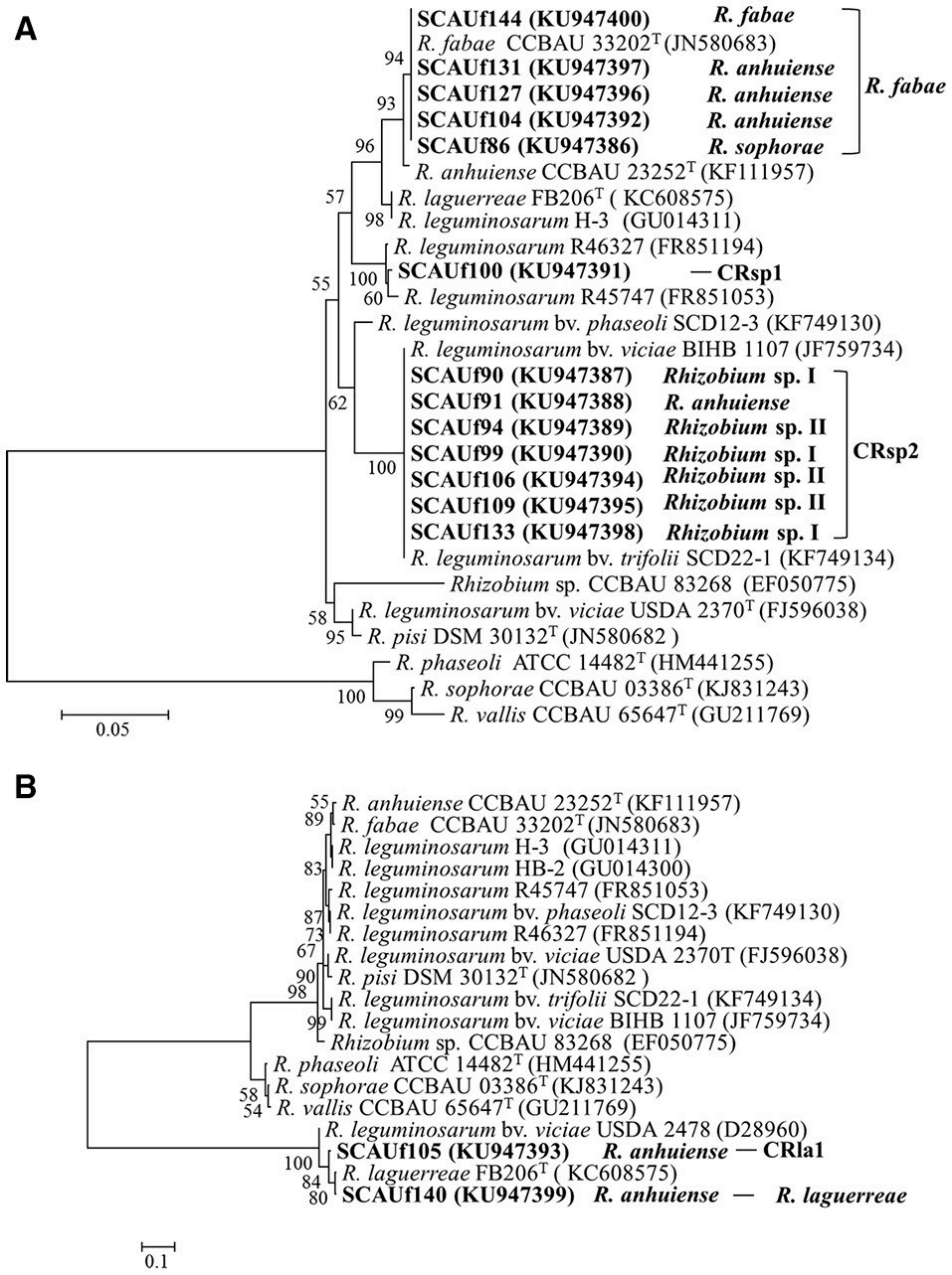
Neighbor-joining tree based on *nodC* gene of 13 representative strains (499 nt) **(A)** and 2 representative strains (230 nt) **(B)** isolated from faba bean (in bold) and reference strains. Genbank accession numbers are in parentheses. Bootstrap values ≥50% are shown on the branches. Scale bar = 5% substitutions per site. *R, Rhizobium*.

Nearly 600 bp *nodC* fragments were amplified from thirteen representative strains using primer pair nodC540/nodC1160. Amplification from strains SCAUf105 and SCAUf140 was successful only while using *R. leguminosarum* sv. *viciae nodC* specific primer pair nodCf/nodCr. Amplification of *nodC* from strains assigned as *Agrobacterium* was not successful. Fifteen strains clustered into five clades in the *nodC* phylogenetic tree (Figure 5, Table 3). The *nodC* of *R. anhuiense* SCAUf131, SCAUf127, and SCAUf104, *R. fabae* SCAUf144 and *R. sophorae* SCAUf86 were 100% similar to that of *R. fabae* type strain (Figure 5A). Similarly to the *nifH* analysis, the *nodC* of *R. vallis* SCAUf100 clustered with *nodC* from non-type strains. Seven strains carried *nodC* 100% similar to that of *R. leguminosarum* non-type strain. The *nodC* of *R. anhuiense* SCAUf140 and SCAUf105 (Figure 5B) were 100 and 97.2% similar to that of *R. laguerreae*.

## Discussion

Due to overcutting and mining Panxi in Southwestern China has suffered serious soil degradation and heavy metal contamination (Xu et al., 2013; Yu et al., 2014). Reclaiming the soils requires sustainable and efficient yet low economic input methods, for example utilization of biological nitrogen fixation (BNF) by legume-rhizobium symbiosis instead of relatively cheap nitrogen fertilizer. To facilitate the utilization of BNF we tested the plant growth promoting ability of rhizobial isolates from faba bean in search of locally adapted, potential inoculant strains. In Ethiopia, chickpea nodulating rhizobia showed big differences in efficiency and nodule numbers, and strains with similar efficiencies did not necessarily induce similar numbers of nodules and vice versa (Tena et al., 2017). Similarly, in our study variations in plant growth promoting ability and nodule numbers were large, and only 11 strains increased faba bean shoot dry mass significantly. Similar to *Leucaena leucocephala* isolates from Panxi (Xu et al., 2013), for many of the strains inoculation resulted in dry mass lower than that in uninoculated plants, highlighting the need to apply selected inocula to promote BNF.

The diversity and identity of the strains were assessed using molecular methods. Faba bean is nodulated by *R. fabae, R. leguminosarum, R. anhuiense, R. laguerraeae* and *A. radiobacter* strains, and the dominant species is different in different regions (Tian et al., 2007, 2008; Youseif et al., 2014; Xu et al., 2015; Zhang et al., 2015; Xiong et al., 2017). In our study, the isolated strains were related to genera *Rhizobium* and *Agrobacterium*. The *Rhizobium* strains were assigned as representing *R. anhuiense, R. fabae, R. sophorae*, and *R. vallis*, and two putative new species in the genus *Rhizobium*. Similar to subtropical provinces in East China (Xiong et al., 2017), *R. anhuiense* was the dominant species among the faba bean nodulating rhizobia in Panxi. To our knowledge, *R. sophorae*, a symbiont of medicinal legume *Sophora flavescens* (Jiao et al., 2015) and *R. vallis*, a symbiont of *Phaseolus vulgaris* (Wang et al., 2011), have not earlier been shown to nodulate faba bean.

The rhizobia-legume symbiosis benefits sustainable agriculture due to the symbiotic nitrogen fixation capacity that needs two key points: nodule infection and nitrogen fixation, both of which need the regulation of symbiosis related genes (Masson-Boivin et al., 2009). In the present study, *nifH* gene that is the structural gene encoding the nitrogenase Fe protein (Masson-Boivin et al., 2009), and *nodC* that is the gene encoding enzymes involved in the synthesis of the core structure of the Nod-factor (Geremia et al., 1994) were selected for sequencing to analyze the symbiotic phylogeny of the faba bean rhizobia in Panxi region. The symbiotic genes are commonly located on a symbiotic plasmid or island which may be transferred (Laranjo et al., 2012; Bakhoum et al., 2014). Faba bean nodulating *R. leguminosarum* strains that carried four different types of nodulation gene *nodD* had all similar *nodC* (Table 4) (Tian et al., 2007). The five types of *nodC* detected in this study suggest higher diversity at symbiosis related gene level. However, considering the six different *nifH*-*nodC* combinations in our study, the faba bean isolates from Yunnan (Tian et al., 2007) and Panxi were approximately equally diverse.

**Table 4:** Rhizobial species, nodD and nodC diversity in subtropical China.

**Province**	**Climate**	**Species**	***nodD* types**	***nodC* types**	**Dominant species**	**References**
Yunnan	Subtropical highland, humid subtropical	5^a^	3^c^	nd	*R. laguerraeae*	Xiong et al., 2017
Yunnan	Subtropical highland, humid subtropical	1^b^	4^d^	1^d^	*R. leguminosarum*	Tian et al., 2007
Anhui, Jiangxi, Henan, Zhejiang	Humid subtropical	3^a^	5^c^	nd	*R. anhuiense*	Xiong et al., 2017
Anhui, Jiangxi	Humid subtropical	2^b^	3^d^	1^d^	*R. leguminosarum*	Tian et al., 2007
Sichuan	Humid subtropical	5^b^	nd	3^e^	*R. leguminosarum*	Xu et al., 2015
Panxi	See Introduction	6^b^	nd	5^e^	*R. anhuiense*	This study

Nd, not determined.

a Determined by amplicon sequencing targeting rpoB.

b Determined by multilocus sequence analysis.

c Determined by amplicon sequencing targeting nodD.

d Determined by RFLP.

e Determined by Sanger sequencing nodC.

The *Desmodium* nodulating rhizobium strains in Panxi region were quite different from those in other places such as temperate and subtropical region of China and Central and North America, possibly due to the special environmental conditions (Xu et al., 2016). The faba bean rhizobia in this area were approximately as diverse as in Sichuan hilly areas and in Yunnan (Table 4) (Xu et al., 2015; Xiong et al., 2017), yet more diverse than in other parts of subtropical China (Tian et al., 2007; Xiong et al., 2017). When looking at the species-*nifH*-*nodC* combinations it is noteworthy that all but two of the six *R. anhuiense* isolates were different. The symbiosis and nitrogen fixation related genes of rhizobia can be transferred laterally (Sullivan et al., 1995). However, whether the increase in diversity in Panxi was caused by lateral transfer cannot be concluded based on our data.

In conclusion, eleven out of 65 faba bean strains in Panxi area could significantly promote plant growth, and were thus considered as potential inoculants. The nodule isolates in this area were diverse belonging to nine species. *R. anhuiense*, the dominant faba bean nodulating species in this area, was diverse both at plant growth promoting ability and symbiosis related gene levels.

## Author contributions

KX and YC conceived and designed the experiments. KX supervised the experiments. YC, LZ, PP, and KX contributed to discussion of the results, and writing and revising the manuscript. LZ performed most of the experiments and analyzed data. QC and CW participated in collecting faba bean nodules and relevant soil information, and relevant meteorological information from the Sichuan meteorological bureau. QL created the map in Figure 1 and revised the manuscript. All authors contributed to writing the article.

### Conflict of interest statement

The authors declare that the research was conducted in the absence of any commercial or financial relationships that could be construed as a potential conflict of interest.
