# Supplementary material for: Faba Bean (Vicia faba L.) Nodulating Rhizobia in Panxi, China, Are Diverse at Species, Plant Growth Promoting Ability, and Symbiosis Related Gene Levels
Source: Front Microbiol. 2018 Jun 20;9:1338. doi: 10.3389/fmicb.2018.01338 (PMC6019463; doi:10.3389/fmicb.2018.01338)

Supplementary Material

Faba bean (*Vicia faba* L.) nodulating rhizobia in Panxi, China, are diverse at species, symbiotic efficiency and symbiosis related gene levels

Y Yuan Xue Chen[1,a], Lan Zou[1,a], Petri Penttinen[2,3,a], Qiang Chen[1], Qi Quan Li[1], Chang Quan Wang[1], Kai Wei Xu[1*]

[1] College of Resources, Sichuan Agricultural University, Chengdu, 611130, China

[2] Zhejiang Provincial Key Laboratory of Carbon Cycling in Forest Ecosystems and Carbon Sequestration, School of Environmental & Resource Sciences, Zhejiang Agriculture & Forestry University, Linan 311300, China

[3] Ecosystems and Environment Research Programme, Faculty of Biological and Environmental Sciences, University of Helsinki, Helsinki, Fin-00014, Finland

[a] These authors contributed equally to this work.

[*]Correspondence: Dr. Kai Wei Xu, xkwei@sicau.edu.cn

Supplementary Figure Legends

Supplementary Fig. S1 Neighbor-joining tree based on multilocus sequence analysis using concatenated sequence of *atpD* (443 nt), *glnII* (580 nt) and *recA* (421 nt) genes presenting the phylogenetic relationship among the representative *Agrobacterium* strains isolated from faba bean (in bold) and reference strains. Bootstrap values ≥ 50% are shown on the branches. Genbank accession numbers are in parentheses. Scale bar = 1% substitutions per site. *A*: *Agrobacterium. A. tumefaciens* genomovar G4=*A. radiobacter*, *A. tumefaciens* G8=*A. fabrum*

Supplementary Fig. S1

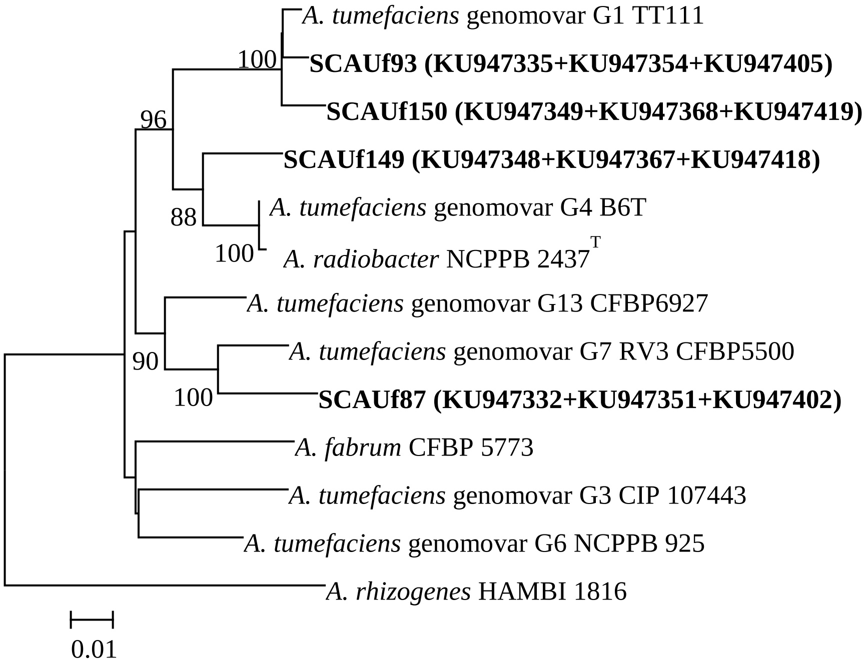

Supplement: Supplementary file 1 [file Presentation_1.PDF]
